# Introducing Pharmaceutical Care to Primary Care in Iceland—An Action Research Study

**DOI:** 10.3390/pharmacy5020023

**Published:** 2017-04-26

**Authors:** Anna Bryndis Blondal, Sofia Kälvemark Sporrong, Anna Birna Almarsdottir

**Affiliations:** 1 Faculty of Pharmaceutical Sciences, University of Iceland, Reykjavik 107, Iceland; 2 Department of Pharmacy, University of Copenhagen, Copenhagen 2100, Denmark; sofia.sporrong@sund.ku.dk (S.K.S.); Denmark; aba@sund.ku.dk (A.B.A.)

**Keywords:** pharmacists, pharmaceutical care, general practitioners, primary care, action research

## Abstract

Even though pharmaceutical care is not a new concept in pharmacy, its introduction and development has proved to be challenging. In Iceland, general practitioners are not familiar with pharmaceutical care and additionally no such service is offered in pharmacies or primary care settings. Introducing pharmaceutical care in primary care in Iceland is making great efforts to follow other countries, which are bringing the pharmacist more into patient care. General practitioners are key stakeholders in this endeavor. The aim of this study was to introduce pharmacist-led pharmaceutical care into primary care clinics in Iceland in collaboration with general practitioners by presenting different setting structures. Action research provided the framework for this research. Data was collected from pharmaceutical care interventions, whereby the pharmaceutical care practitioner ensures that each of a patient’s medications is assessed to determine if it is appropriate, effective, safe, and that the patient can take medicine as expected. Sources of data included pharmaceutical care notes on patients, researcher’s notes, meetings, and interviews with general practitioners over the period of the study. The study ran from September 2013 to October 2015. Three separate semi-structured in-depth interviews were conducted with five general practitioners from one primary health care clinic in Iceland at different time points throughout the study. Pharmaceutical care was provided to elderly patients (*n* = 125) before and between general practitioners’ interviews. The study setting was a primary care clinic in the Reykjavik area and the patients’ homes. Results showed that the GPs’ knowledge about pharmacist competencies as healthcare providers and their potential in patient care increased. GPs would now like to have access to a pharmacist on a daily basis. Direct contact between the pharmacist and GPs is better when working in the same physical space. Pharmacist’s access to medical records is necessary for optimal service. Pharmacist-led clinical service was deemed most needed in dose dispensing polypharmacy patients. This research indicated that it was essential to introduce Icelandic GPs to the potential contribution of pharmacists in patient care and that action research was a useful methodology to promote and develop a relationship between those two health care providers in primary care in Iceland.

## 1. Introduction

The pharmacy profession has increasingly been making individualized care plans for patients [1] since Hepler and Strand described the term pharmaceutical care practice in 1990 [2]. Pharmaceutical care represents a patient-centered approach where the pharmacists, in collaboration with other health care professionals, are responsible for patients’ drug therapy. The purpose is to achieve positive outcomes intended to improve the patient's quality of life. The pharmaceutical care practitioner ensures that each of a patient´s medications (prescription, nonprescription, alternative, or traditional medicines) is assessed to determine if it is appropriate, effective, safe, and that the patient is able to take medicine as expected [3,4,5]. The service can be provided in different settings of care; pharmacies, primary, secondary, and tertiary care [6,7]. Sometimes, the pharmaceutical care practitioners are employed in the primary care clinics and practice full-time or part-time in the clinic providing this service [8]. Other pharmaceutical care practitioners are located outside of the primary care clinic in the community pharmacy [9], in the hospital setting [10], or as self-employed consultants [9]. Even though reliable data have pointed out that pharmaceutical care can lead to progress in health outcomes and cost-effective therapy [11], and positive impact on Health-Related Quality-of-Life Outcomes [12], the implementation has not been as fast as one might have expected [13,14]. Lack of time and attitudes/opinions of other professionals, clinical education, communication skills, and remuneration have been reported as barriers to implementation. It should be noted that barriers are not the same in all countries [15,16,17,18].

In Iceland, pharmaceutical care services are provided in hospitals but not in other settings of care, despite the requirement for pharmaceutical care provision being written into legislation in 1994 [19]. The main challenge in Iceland is that, currently, there is little communication between community pharmacists and general practitioners (GPs) on clinical issues [20], even though globally pharmacists are taking on a larger role as primary care patient care providers [1]. GPs do not recognize pharmacists as health care providers, nor do they have any experience with pharmacist-led clinical services [20]. International studies abound on the impact of clinical pharmacy services on patient outcomes [21] and some interventions have focused on pharmacist–GP cooperation [22]. To date, studies have not focused on the actual process when pharmacists develop an outpatient service, where none exists, in close collaboration with GPs.

The aim of action research methodology is to facilitate continuous improvement and involvement [23]. Since the implementation of pharmaceutical care is shown to be challenging, this approach was chosen for this study. It was also hoped that it could contribute to making the development and implementation as successful as possible. Therefore, the aim of this study was to use action research methodology to introduce and study pharmacist-led pharmaceutical care in primary care in collaboration with GPs and to test this model in different settings with the goal of meeting specific local and Icelandic needs.

## 2. Materials and Methods

### 2.1. Research Design

The origins of action research are often credited to the social psychologist Lewins at the end of the Second World War [24]. Reason and Bradbury [25] describe action research as a “family of approaches” where both quantitative and qualitative research methods are used. It is based on a different paradigm from other research because traditionally the researchers do a study on people but action researchers do research with people [26]. The action research process focuses on action and change. The overall characteristic of action research is the use of cycles aiming to meet identified needs. The process steps are: diagnosing and analyzing problems, planning, implementing/taking action, and evaluating. After the evaluation, a new cycle can start based on the new situation [25,26,27].

In this case, the problem was how to introduce and adapt the pharmaceutical care service into the Icelandic primary care clinics. Action research was used as it was expected that several cycles would be needed to get to a process that was feasible for the existing organization and at the same time providing benefit to patients. By involving and making GPs active in decisions about the implementation, it was expected that they would be more willing to accept pharmacist-led pharmaceutical care. An active participation strategy was used; the first author was active in introducing and starting the service, i.e., being a practitioner, while at the same time being a researcher. The process started by understanding GPs’ perspectives and introducing a service, which was then modified throughout the action research cycles.

### 2.2. Setting

The study settings were one primary care clinic in the Reykjavik area and homes of patients who received the pharmacist-led pharmaceutical care. In Iceland, publicly funded primary care clinics were established four decades ago offering services to the whole population. They are intended to be the patient´s first point of contact to the health care system, although GPs do not have a formal gatekeeping role. The primary care clinics offer various health and nursing services, general medical service, general nursing care, infant and maternity service, school nursing, vaccinations for adults, and other services. They are located all around the country and offer different kinds of service depending on location [28]. The primary care clinic participating in the research is situated in the Reykjavik area and provides all of the services mentioned above.

Five GPs from the primary care clinic (three females and two males) and 125 of their patients participated in the action research process. The primary care clinic GPs chose participants over 65 years of age based on the risk criteria established by the Home Medicines Review (HMR) program in Australia [29]. GPs then made a referral to the pharmacist for pharmaceutical care service. The choice of criteria was based on that they have shown works well in identifying patients in need of pharmaceutical care [29] and that they were well accepted by GPs.

### 2.3. Data Collection and Analysis

Data was collected from pharmaceutical care interventions with patients, research notes, meetings, and in-depth interviews with GPs throughout the study, which ran from September 2013 to October 2015.

Pharmaceutical care intervention: The researcher provided pharmaceutical care as defined in the pharmaceutical care literature [3,4,5] to 125 patients throughout the study. Here the pharmaceutical care practitioner ensured that each of a patient´s medication was assessed to determine if it was appropriate, effective, safe and that the patient was able take medicine as intended. Twenty-five patients received care in a pilot phase, 50 elderly home-dwelling patients in the first round of the action research process with no access to their medical records. In the second round, 50 elderly home-dwelling patients receiving dose dispensed medicines received pharmaceutical care services at the primary care clinic where the pharmacist had access to the patients’ medical records.

In-depth interviews with GPs: Three in-depth interviews with each of the five GPs were conducted. Interviews were audio-recorded, transcribed verbatim, and thematically analyzed independently by the first and last authors. Themes were discussed among the researchers for agreement. Coding and thematic analysis were undertaken using conventional content analysis [30] and NVivo 11. Data were evaluated in the Icelandic (original language) and relevant quotes were translated into English. The first round of in-depth interviews studied the GPs’ perspectives on various issues, the past decade’s development of primary care in Iceland, today’s status regarding medicine use and monitoring, GPs’ use and perception of pharmacists, and their vision for primary care in the future. Specifics were sought regarding the pharmaceutical care service, the GPs’ perceptions of the pharmacist-provided pharmaceutical care at the outset, their views on possible patient health outcomes, resource utilization, ways of collaborating, the pharmacist’s physical location, and remuneration for the service. In the second round, GPs were asked about the pros and cons of the service provided and their views on collaboration with the pharmacist on clinical issues. Also, some practical matters such as the structure of the report given to them by the pharmacist and how they chose patients in the research. In the third round of interviews, GPs were asked the same questions as in the second round. Additionally, their views on differences between the two ways of providing pharmaceutical care, their ideas about the best way to provide this service in primary care in the future and their current opinion of pharmacists and their role in patient care were asked for.

Meetings: Throughout the duration of the project, three meetings were held between the participating pharmacist and the participating GPs. The meetings were conducted to explore the progress of the project and to find common ground on which to move forward. The meetings were not recorded, but notes were taken and minutes were written up including ideas, discussions, and decisions made.

Research notes: Throughout the entire project, the participating researcher kept notes of her experience. These notes consist of descriptions of events to document the project´s process and progress. These notes supplemented the data and were used to further understand the project, interventions, GP meetings, and interviews. The participating researcher continuously reflected on the data by speculating on the issues and what had been planned, discovered, and achieved during the process.

### 2.4. Ethical Approval

Ethical approval (Nr. 12-218) was obtained from the National Bioethics Committee and The Data Protection Authority in Iceland.

## 3. Results

### 3.1. Description of Action Research Cycles

**Figure 1:**
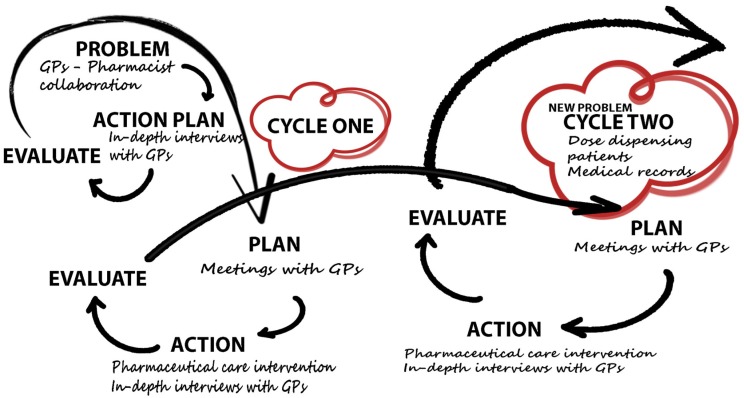
Overview of the two action research cycles in the study.

Throughout the process of the study period, one action led to another, and two cycles emerged as shown in Figure 1.

The cycles are described in detail below, but Table 1 provides an overview of the process.

### 3.2. Cycle One

#### 3.2.1. Diagnosing the Problem

The first round of interviews while planning cycle one showed several unmet needs regarding medicines and patient monitoring in the Icelandic health care system. GPs suggested ways in which these gaps could be addressed and pharmacist-led pharmaceutical care was one of the suggestions. The GPs’ communication with pharmacists in the primary care setting mostly surrounded practical issues—not clinical.

They were not familiar with pharmaceutical care service, but due to polypharmacy and various other drug-related problems, they found that pharmacists should be more involved in patient care.

#### 3.2.2. Plan

A meeting was held with GPs participating in the study to find common ground moving forward with the study based on the results of diagnosing the problem. The results of the meeting were to introduce pharmacist-led pharmaceutical care service to GPs personally and then interview them immediately after. The focus was put on elderly home-dwelling polypharmacy patients, and the participating researcher had no access to patients’ medical records.

#### 3.2.3. Action

The participating researcher provided pharmaceutical care to 50 elderly home-dwelling polypharmacy patients with no access to medical records. Then, she interviewed participating GPs.

#### 3.2.4. Evaluation/Reflection

Results from the second round of GP interviews revealed that GPs found the service useful, but at the same time they found it needed to be more structured. After the pharmacist had met with the patient, the doctor did not see him/her subsequently, and therefore the doctors did not review the pharmacist’s report immediately. It was pointed out that “this administration takes too much time, call the patient and get a hold of him you understand this will increase our work so I would like it to be more streamlined” (Interview 2, Participant 3). 

GPs thought pharmacists’ education should be concerned more with providing patient care. Because GPs had difficulties reviewing patients’ drug lists on a daily basis, they found themselves needing the pharmacist’s clinical service. One doctor mentioned, “In the daily work at the clinic you don’t have time [to review the patient’s medication list] because of too many tasks, and too few GPs [being] employed [at the clinic]” (Interview 2, Participant 4). Due to structural issues, they thought the service was most needed for dose dispensed polypharmacy patients. In Iceland patients who get their medications dose dispensed are prescribed the medicines on a one-year basis. One doctor said, “Today, dose dispensed patients are more likely to be automatically prescribed than others” (Interview 2, Participant 1).

The researcher as a pharmaceutical care provider identified the need for medical records providing this service. Many of the pharmacist’s comments could be omitted if clinical information on the patient was available. Also, in the researcher’s opinion, the distance between her and the GPs led to fewer communications crucial for the proper pharmaceutical care of patients.

### 3.3. Cycle Two

#### 3.3.1. Plan

A second meeting with the GPs was held to explore the progress and to find common ground to move forward with the program. The GPs found themselves needing the pharmacist’s service in dose dispensing polypharmacy patients due to structural issues and also because they found the service process needed to be more explicit. From the pharmacist’s point of view, there was a lack of communication as well as the lack of access to medical records. Therefore, it was decided that the participating researcher would provide pharmaceutical care to dose dispensing patients at the primary care clinic with access to medical records and then interview GPs immediately after.

#### 3.3.2. Action

The participating researcher provided pharmaceutical care to 50 dose dispensing patients at the primary care clinic with access to medical records. She then interviewed participating GPs on the pharmacist service provided.

#### 3.3.3. Evaluation/Reflection

##### GPs’ view of pharmacists as health care providers:

It was clear in the third round of interviews that the GPs had much more to say about pharmacists than at the beginning of the project and their comments were more precise and to the point. All participants thought highly of pharmacists, even though they had little or no connections with pharmacists regarding clinical issues. They agreed that seeing the pharmacist in practice increased their positive view of pharmacists. One doctor said; “I’m happy that they are focusing more on consumers that patients are important not just the pills” (Interview 3, Participant 3). Another said; “This project has increased my positive attitude towards pharmacists” (Interview 3, Participant 4). Yet another GP said, “I have to answer this in the affirmative now that I have seen how you work [as a pharmacist]” (Interview 3, Participant 1).

##### Pharmacist-led pharmaceutical care:

At the end of the study process, GPs had experienced pharmacist-led pharmaceutical care service personally in two different settings. All GPs thought it was a good quality control on prescribing and they did not have any negative attitudes towards the service provided. The only challenge remaining was the lack of time spent reviewing patient reports from the pharmacist. They found the service to be useful and pointed out the pharmacists’ comments about interactions, adverse drug reactions, and indications as very helpful input into clinical decision-making. Also, they appreciated getting tips regarding drugs that are rarely used. The input demanded by the GPs from the pharmacist changed significantly over the study period. At the beginning of the project, the GPs focused mostly on the pharmacist providing information about interactions, but by the end, other issues were considered, as mentioned above. One experienced doctor said “This [pharmacist service] is a new angle when you look at pharmacist involvement in patients’ medicines, and it is real progress and a quality improvement that lifts up the operation of the primary care clinic” (Interview 3, Participant 4). Another said “I think this [pharmacist service] is great. There is information that I have received that I had not been aware of or didn't know about, so you learn a lot” (Interview 3, Participant 5).

All GPs found the service helpful and agreed that it would, in the long-term, facilitate the GPs’ practice. One GP summed it up “We have a lot of patients who see different doctors and are consuming lots of drugs, and we [as GPs] have difficulties reviewing whether all the drugs are necessary and so on” (Interview 3, Participant 5).

##### The future of GP-pharmacist collaboration in Iceland:

All GPs participating in the research openly stated that they felt pharmacists should work at the primary care clinic and be a part of the healthcare team as a medicines expert. When analyzing their comments from interview one to three, and focusing on the differences between the interventions, it was clear that they were more pleased with the last intervention, which took place at the primary care clinic. The main reason stated by all participants was because of the face-to-face communication. One GP said “I think that the presence of these health professionals such as doctors and pharmacists that you recognize faces and you have spoken to each other is priceless. So, working at a close distance between these health professionals is a good thing” (Interview 3, Participant 4).

GPs mentioned other practical issues such as pharmacist access to medical records, pharmacists having other roles working at the clinic (such as educating other health care providers), and the pharmaceutical care service needing to be well structured and streamlined to have benefits. However, they had all throughout the process mentioned the importance of the face-to-face communication.

## 4. Discussion

This study aimed to introduce and subsequently study pharmacist-led pharmaceutical care in primary care in collaboration with GPs using action research methodology. The main findings of this study are that GPs’ knowledge about pharmacists as patient care providers developed during the research period. Second, GPs found it better when pharmacist and GPs worked side by side in clinical decision-making, and they would like to have access to a pharmacist on a daily basis. Lastly, pharmacist access to medical records is necessary for an optimal service.

Since pharmacists are not always considered as patient care providers in contrast to nurses and doctors, it is important to introduce pharmacist-led clinical services in a systematic manner. It can be assumed that, to implement a new health care service successfully, the service has to be needed not by the provider (the pharmacist) but by others, for example, groups such as patients, doctors and nurses. These groups cannot demand services that they do not recognize as necessary. The main challenge regarding pharmacist-GP collaboration in Iceland is that GPs neither know pharmacists as health care providers nor their clinical services [20]. This study was the first in introducing pharmacists as potential patient care providers in an established primary care clinic in Iceland. The results show that the more GPs are familiar with the service, the more they find it useful. This particular result has been reported by other researchers [31]. In addition, developing a trusting relationship is an important factor for efficient collaboration [32,33]. In our study, the pharmacist established credibility as a team member by working as a patient care provider and answering drug-related questions. Other studies have shown that pharmacists working with physicians as an information resource has established credibility [34]. Another crucial part is that the pharmacist service needs to be well structured to benefit GPs. Giving them an opportunity to structure the service in an action research process with the pharmacist, enhanced their buy-in and appreciation of the new service. To build a good interdisciplinary unit, it is important that participants identify the role, purpose, and a clear vision for the team [35]. Due to the GPs’ current heavy workload, and since this is a new service to them, they did not at the outset fully understand the role of the pharmacist. This research was initiated to remedy that.

It was surprising that GPs wanted the participating researcher to focus the clinical effort on dose dispensed patients since they already receive a simple medication review (based on the medication history in the pharmacy) by a pharmacist. However, a systematic review by Sinnemäki et al. [36] revealed that dose dispensing patients are more at risk of having the drug treatment unchanged for an extended period. Possible reasons are likely—as the GPs in this research pointed out—that dose dispensed patients are often prescribed medicines for a long time without a GP or a clinical pharmacist reviewing their medication list.

Another issue, which is important to consider, is various locations and needs for the service. Since pharmacist clinical service is new in primary care in Iceland, it is not possible to have a pharmacist in every clinic due to lack of pharmacists working in patient care. Therefore, it is necessary to make an implementation plan taking into account factors such as the number of elderly patients, the prevalence of polypharmacy, the primary care clinic structure, and which clinics are in most need of pharmacist service. USA, Canada, and the UK are examples of countries that have integrated pharmacists into primary care [37,38,39].

A single action research study, which introduces pharmacist-led pharmaceutical care in collaboration with GPs, is not sufficient to get clinical pharmacy services on track in Iceland. As mentioned above, the literature pointed out different barriers to implementation, such as lack of time and attitude/opinion of other professionals, clinical education, communication skills, and remuneration [15,16,17,18]. In order to successfully roll out the service to other clinics in Iceland, a workforce plan taking our findings and all those other factors into consideration is needed.

By using action research methodology, the research was able to adapt to the context and setting, making the success of implementation more probable. Those aspects were crucial in this study because it allowed the project to constantly develop in the primary care clinic and to change along the way. Also, by collaborating with the GPs, different perspectives were presented, and this increased the project’s validity and efficiency.

This study has a few limitations worth mentioning. First, in action research, the close relationships between the researcher and participants have been criticized, which can easily introduce biases. Conversely, it has been reported that this closeness can result in more valid data [40]. This can also be a limitation in the data analysis. However, two researchers not directly involved in the process (the second and third authors) also analyzed and interpreted the data. When introducing a healthcare profession in the team, it is vital for this professional to collaborate with other team members [35]. In Iceland, doctors and nurses are currently the only health professions that play a significant role in the primary care clinics with very little input from other health care professions. Therefore, the second limitation of our study is that nurses were not involved in the project. Third, action research can be time-consuming and a few months are often insufficient duration to establish a permanent connection and trust, although in this case, the pharmacist managed to build trust with the GPs and staff during this period. Last, because this research took place on a local scale, it cannot be generalized to a broader context. Nonetheless, the process and the results of it can be an inspiration for other settings.

## 5. Conclusions

When implementing pharmaceutical care practice, many barriers have been noted in the literature. In Iceland, the lack of communication between GPs and pharmacists was one of them. This research indicated that action research is a useful methodology to promote and develop a relationship between those two health care providers in primary care. The most efficient collaboration is when pharmacists and GPs work side by side at the primary care clinic.

## Figures and Tables

**Table 1:** Overview of the two action research cycles. The time period and stage within each cycle are shown, and for each stage, the specific objectives, process, and output leading to the subsequent stage.

Time Period/Cycle Stage	Objective	Process	Output
September to December 2013 Diagnosing the problem cycle one	Understand GPs’ perspective on various issues	Conduct and analysis of the first round of in-depth interview with participant GPs	There are several unmet needs regarding medicines and patient monitoring, and GPs are not familiar with pharmacist clinical service.
January 2014 Plan cycle one	Find common ground to move forward with the program from the results	Meeting with participating GPs	GPs are unfamiliar with pharmacist services. It was decided to provide pharmaceutical care to 50 patients and focus on elderly home dwelling polypharmacy patients and then interview GPs immediately after.
February to October 2014 Action cycle one	Provide pharmaceutical care and focus on polypharmacy patients	Pharmaceutical care process as defined by Cipolle et al. [3,4,5]	The participating researcher provided pharmaceutical care to 50 elderly home-dwelling patients with no access to medical records.
November and December 2014 Action cycle one	Get GPs views on the pharmacist service provided	Conduct of the second round of in-depth interviews with GPs	The second round of in-depth interviews with participating GPs.
December 2014 and January 2015 Evaluation/Reflection cycle one	Describe the results from the pharmaceutical care intervention and in-depth interviews	Analysis of the second round of interviews with GPs and research notes	GPs found the pharmaceutical care service useful but that it needed more structure. They found the service most needed in dose dispensing polypharmacy patients. Pharmacist needed medical records and increased contact with GPs to provide proper pharmaceutical care service.
January 2015 Plan cycle two	Find common ground to move forward with the program	Meeting with participating GPs	It was decided to provide pharmaceutical care to 50 dose dispensing patients at the primary care clinic with access to medical records and then interview GPs immediately after.
February to June 2015 Action cycle two	Provide pharmaceutical care and focus on polypharmacy dose dispensing patients	Pharmaceutical care process as defined by Cipolle et al. [3,4,5]	The participating researcher provided pharmaceutical care to 50 patients. The service was provided at the primary care clinic with access to medical records.
June 2015 Action cycle two	Get GPs’ views on the pharmacist service provided	Conduct of the third round of in-depth interviews with GPs	The third round of in-depth interviews with participating GPs.
August to October 2015 Evaluation/Reflection cycle two	Describe the result from the pharmaceutical care intervention and in-depth interviews	Analysis of the third round of interviews and research notes	GPs found this second type of intervention to be an improvement and that it gave useful input into clinical decision-making. Direct contact between the pharmacist and GPs is increased when working in the same physical space. Pharmacist’s access to medical records is necessary for optimal care.
